# The impact of the metabolic phenotype on thyroid function in obesity

**DOI:** 10.1186/s13098-016-0177-x

**Published:** 2016-08-24

**Authors:** Paolo Marzullo, Chiara Mele, Stefania Mai, Gabriele Guzzaloni, Davide Soranna, Maria Antonella Tagliaferri, Maria Elisa Berselli, Flavia Prodam, Daniela Surico, Gianluca Aimaretti, Massimo Scacchi

**Affiliations:** 1 Division of General Medicine, I.R.C.C.S. Istituto Auxologico Italiano, Ospedale S. Giuseppe, Casella Postale 1, 28921 Verbania, Italy; 2 Laboratory of Metabolic Research, I.R.C.C.S. Istituto Auxologico Italiano, Ospedale S. Giuseppe, 28921 Verbania, Italy; 3 Division of Biostatistics, Epidemiology and Public Health, Department of Statistics and Quantitative Methods, University of Milano-Bicocca, 20126 Milan, Italy; 4 Endocrinology, Università del Piemonte Orientale, 28100 Novara, Italy; 5 Division of Obstetrics and Gynaecology, Department of Translational Medicine, Università del Piemonte Orientale, 28100 Novara, Italy; 6 Department of Clinical Sciences and Community Health, University of Milan, 20122 Milan, Italy

**Keywords:** Thyroid, Obesity, Diabetes mellitus, Metformin

## Abstract

**Background:**

Obesity is known to promote mild hyperthyrotropinaemia by unknown metabolic mechanisms. This investigation aimed to explore the association between thyroid function and metabolic phenotype in euthyroid obese individuals. Retrospective, cross-sectional study. Tertiary care center.

**Methods:**

952 euthyroid obese individuals referred to our Institution for obesity. Serum levels of TSH, FT4, glucose, insulin and HbA1c levels, lipid profile, liver function and proinflammatory indices were measured. Resting energy expenditure was assessed by indirect calorimetry and body composition by bioimpedance analysis.

**Results:**

On admission, 306 patients had previously diagnosed diabetes mellitus on treatment with metformin, while 113 patients were diagnosed with incident diabetes mellitus. Serum TSH levels were similar between metformin-treated diabetic subjects and metformin-untreated subjects, while FT4 was slightly but significantly higher in the former. Analysis stratified by TSH categories found no effect of metformin-treated diabetes mellitus on TSH levels. Interestingly, obese patients with incident diabetes showed lower TSH levels than normoglycaemic ones. In correlation studies on the whole dataset, an association related TSH to BMI and total cholesterol levels, which was lost upon adjustment for individual confounders. FT4 levels were found to be inversely related to BMI, insulin resistance and triglycerides, while being directly associated with HDL-cholesterol levels. These correlations remained unaltered after controlling for individual confounders. In multivariate linear regression analysis, TSH was associated with FT4, total cholesterol and BMI values. Significant predictors of FT4 were constituted by previously diagnosed diabetes mellitus, BMI, TSH and age.

**Conclusions:**

In euthyroid obese subjects, FT4 seems more closely related than TSH levels to parameters of cardiometabolic risk. TSH levels did not differ between metformin-treated and untreated subjects, while they were lower in patients with incident diabetes mellitus compared to normoglycaemic ones.

## Background

Thyroid disorders impact body weight in multiple ways, and hypothyroidism is traditionally claimed as a contributor of weight accrual via altered metabolic efficiency, water retention, decreased lipolysis [1]. Nonetheless, case-control studies and epidemiological surveys revealed that TSH levels tend to follow body weight accrual as well as development of obesity and insulin resistance, independent of hypothyroidism [2–9]. The causal mechanism underlying this link remains unidentified, yet a compensatory response operated by the hypotalamo-pituitary unit via leptin and directed to counteract weight accrual, is very likely involved [1, 10].

Obesity, type 2 diabetes mellitus (T2DM) and thyroid diseases are the most frequent endocrine disorders and often coexist in the same individual [11]. Following an original case study [12], cross-sectional and prospective studies have shown that the antidiabetic drug metformin can decrease TSH levels in patients with subclinical and/or overt hypothyroidism, while other antidiabetic agents do not yield such effect [13–17]. In subsequent studies, it has been clarified that metformin can alter TSH levels also in euthyroid subjects if TSH levels lay in the mid-high normal range (i.e., >2.5–3.0 mIU/L) [18–20].

Considering the metabolic derangement commonly associated with obesity as well as the frequent use of metformin in overweight patients owing to its insulin-sensitizing effect and potential reduction of body weight [21], we aimed at assessing the impact of the metabolic phenotype, metformin-treated diabetes mellitus, and incident diabetes mellitus on thyroid function in euthyroid obese patients, and thus further investigating the potential thyroidal determinants of cardiometabolic risk in this setting.

## Methods

This study enrolled 952 obese patients [M/F = 410/542, median age 58 (IQR 52–66) years, median BMI 45 (IQR 41.4–49.4) kg/m^2^] referred to our institution for work-up and rehabilitation of obesity and its comorbidities. Subjects included in the current study were participants of the TONDO study (T2DM of new diagnosis in obesity), an ongoing study initiated in 2012 and designed to investigate the relationship between glucose abnormalities and indices of organ damage in subjects with uncomplicated and complicated obesity [22], and were included according to inclusion criteria and availability of complete thyroid function assessment. Written consent was obtained from all patient, after full explanation of the purpose and nature of the study. The investigation was approved by the local ethical committee, functioning according to the 3rd edition of the Guidelines on the Practice of Ethical Committees in Medical Research. The current cohort included: (1) 646 euthyroid obese subjects [group OB-Eu; males/females = 283/363, median age 55 (IQR 48–65) years, BMI 43.7 (IQR 40–48.9) kg/m^2^], 113 of whom were diagnosed on admission with T2DM based on fasting glycaemia and/or HbA1c levels; (2) 306 euthyroid obese subjects with T2DM on treatment with metformin for at least 6 months [group OB-EuM; males/females, 127/179; median age 58 (IQR 50–65) years; BMI 45.6 (IQR 41.4–49.6) kg/m^2^; median diabetes duration 8.1 years (IQR 6.9–9.4)]. Exclusion criteria were age below 18 or above 90 years; previous or current use of medications potentially interfering with thyroid function (such as amiodarone, steroids or lithium carbonate therapy); previous or current treatment with levo-thyroxine; known autoimmune diseases (including thyroid) and/or hypothyroidism; type 1 diabetes mellitus; pregnancy; liver or kidney disease. In case of TSH value >4.5 mIU/L, a condition not mandatorily indicative of thyroid dysfunction in obesity, patients underwent a preliminary thyroid screening inclusive of re-analysis of TSH and measurement of fT3, fT4 and anti-thyroid antibody titer, to exclude true thyroid hypofunction. Screening test included glucose and insulin levels, lipid profile, indices of liver function and proinflammatory state, in fasting conditions. Patients were not prescribed diet therapy, dietary supplements, or antiobesity compounds for at least 3 months prior to entering the study.

### Body measurements

All subjects underwent body measurements wearing light underwear, in fasting conditions after voiding. Weight and height were measured to the nearest 0.1 kg and 0.1 cm, respectively. BMI was expressed as body mass (kg)/height (m)^2^. Obesity was defined for any BMI over 30 kg/m^2^ [23]. Waist circumference was measured midway between the lowest rib and the top of the iliac crest after gentle expiration; hip was measured as the greatest circumference around the nates. Anthropometric data were expressed as the mean of two measurements.

Fat mass and free fat mass, expressed as percentage of total body mass, were assessed by bio-impedance analysis (BIA, 101/S Akern; Florence, Italy) the morning after overnight fasting and after voiding. The two vector components of impedance (i.e. resistance and reactance) were obtained by single measurements; before each testing session, the external calibration of the instrument was checked with a calibration circuit of known impedance value. The mean coefficient of variation was 1 % for within-day and 3 % for weekly intraindividual measurements in the steady-state condition in either site and 2 % for interoperator variability.

Resting energy expenditure (REE) was expressed in kilocalories per 24 h and determined in a thermoregulated room (22–24 °C) by computed open-circuit indirect calorimetry, measuring resting oxygen uptake and resting carbon dioxide production by a ventilated canopy (Sensormedics, Milan, Italy) at 1-min intervals for 30 min and expressed as 24 h value. Predicted REE was calculated by the Harris–Benedict formula and allowed to test for metabolic efficiency.

### Laboratory tests

Insulin resistance was calculated by the homeostatic model of insulin resistance (HOMA-IR) index: insulin (mIU/L) × [glucose (mmol/L)/22.5]. A HOMA-IR value greater than 2.0 was considered indicative of insulin resistance, as obtained in a sample of the Italian population [24]. The homeostatic model of β cell function (HOMA-B) was used to describe the functionality of pancreatic beta cells and calculated using the following formula: 20 [insulin (mIU/L)/glucose (mmol/L) − 3.5]. HbA1c levels were determined in 290 patients of the metformin-untreated group and all metformin-treated patients. ADA recommendations for 2012 [25] were used for the definition of glucose metabolism and T2DM based on fasting plasma glucose (FPG) and glycated hemoglobin (HbA1c), as follows: normal FPG if <5.6 mmol/L; impaired FPG (IFG) if FPG was 5.5–6.9 mmol/L; T2DM if FPG was ≥7.0 mmol/L on 2 days apart. HbA1c values of 5.7 and 6.5 % were considered as the threshold of normal glucose metabolism and T2DM, respectively. Undiluted serum samples were assayed for fT4 and TSH using an automated chemiluminescence assay system (Immulite 2000; DPC, Los Angeles, CA). The principle of the method is a two-site, solid-phase chemiluminescent immunometric assay or competitive immunoassay. Normal values for TSH are 0.4–4.5 mIU/L, and for fT4 102.9-244.5 nmol/L. Insulin levels were measured by immulite. Glucose, total cholesterol, high-density (HDL) and low-density lipoprotein (LDL) cholesterol, and triglycerides were measured by enzymatic methods (Roche Diagnostics, Mannheim, Germany). Fibrinogen levels were determined with the Clauss methodology by Hemosil assay (IL Coagulation System, Instrumentation Laboratory, Bedford, MA). Ultrasensitive C-reactive protein (CRP) was measured by CRP (latex) HS Roche kit, having sensitivity of 0.03 nmol/L, intraassay and interassay CVs of 2.51–5.35 and 4.25–5.79 %, respectively, as reported by the manufacturer. Alkaline phosphatase, aspartate aminotransferase (AST) and alanine aminotransferase (ALT) were assayed according to the International Federation of Clinical Chemistry (IFCC), without pyridoxal-5′-phosphate, using the Cobas Integra 800 (Roche Diagnostics). Gamma-glutamyltranspeptidase (GGT) was measured by enzymatic colorimetric test using Roche/Hitachi 904/911/912/917/modular (Roche Diagnostics).

### Statistics

Statistical analysis was performed using SPSS version 18 (Somers, NY, USA) on log transformed data to correct for the non-Gaussian distribution obtained by the Shapiro–Wilk test. Values are expressed as medians and interquartile ranges. Mann–Whitney test was used for comparison between subgroups. Spearman’s correlation analysis and the Chi square were used to identify significant associations between variables of interest. The role of non-collinear variables on TSH and fT4 levels was tested by stepwise multiple regression analysis using as independent variables age, gender, BMI, use of metformin, total cholesterol and fT4 or TSH, depending on whether TSH or fT4 levels were analyzed, respectively. HOMA-IR and waist circumference were employed as additional covariates in this model after exclusion of potential collinear variables. Logistic regression analysis to test the effect of metformin-treated diabetes mellitus and other variables of interest on the levels of thyroid parameters. Statistical significance was set at 5 %.

## Results

A summary of anthropometric and biochemical data are reported in Table 1. BMI values were comprised between 30–73.6 kg/m^2^, and were >40 kg/m^2^in 78.8 % (750 patients), >35–39.9 kg/m^2^ in 17.8 % (172 patients), and >30–34.9 kg/m^2^ in 3.1 % of cases (30 patients). The metformin-treated diabetic subgroup exhibited greater BMI, waist circumference and fat mass than their counterpart. Both subpopulations were, however, severely insulin resistant and HOMA-IR exceed the normal threshold of 2 [23] in 81.6 % of metformin-untreated and 87 % of metformin-treated diabetic patients. Ninety-four metformin-treated diabetic subjects were on statins at the time of the study, which likely explained the differences in total and LDL-cholesterol noted between groups.

**Table 1:** Summary of anthropometric and biochemical data obtained in the whole study population, in the euthyroid metformin-treated obese diabetic subpopulation (OB-EuM) and the euthyroid obese metformin-untreated subpopulation (Ob-Eu)

Variables	Whole population (n = 952)	OB-EuM (n = 306)	Ob-Eu (n = 646)	p
Males/females	410/542	127/179	283/363	0.5
Age (years)	56 (49–65)	58 (50–65)	55 (48–65)	0.07
BMI (kg/m^2^)	44.4 (40.5–48.9)	45.6 (41.4–49.6)	43.7 (40.0–48.7)	0.003
Weight (kg)	117.8 (102.8–133.5)	118.3 (106.0–134.0)	117.3 (101.6–133.1)	0.09
Height (cm)	161.8 (155–171)	161 (155–170)	162 (155–171)	0.3
Waist (cm)	129 (119–138)	131 (122–140)	127 (116–137)	<0.001
Waist-to-hip ratio	0.96 (0.89–1.92)	0.97 (0.91–1.04)	0.94 (0.88–1.02)	<0.001
REE (kcal/day)	1909 (1651–2247)	1963 (1740–2280)	1884 (1598–2233)	0.003
Fat mass (%)	46.3 (41.0–51.8)	47.3 (41.9–52.6)	45.7 (40.4–51.3)	0.009
Body water (%)	39.7 (36.2–44.2)	39.5 (36.3–43.9)	39.8 (36.2–44.4)	0.7
TSH (mIU/L)	1.72 (1.21–2.47)	1.72 (1.19–2.45)	1.72 (1.22–2.47)	0.7
fT4 (nmol/L)	146.3 (132.5–162.1)	151.9 (135.1–163.5)	145.4 (131.3–162.2)	<0.001
Insulin (pmol/L)	100.7 (65.9–145.8)	98.6 (62.5–150.0)	102.1 (68.1–143.1)	0.5
Glucose (mmol/L)	5.77 (5.05–7.21)	7.38 (6.05–8.99)	5.38 (5.38–6.22)	<0.001
HbA1c (%)	6.6 (6.0–7.9)	7.5 (6.5–8.7)	6.1 (5.7–6.7)	<0.001
HOMA-IR	4.0 (2.5–6.1)	4.7 (2.9–7.2)	3.7 (2.3–5.6)	<0.001
HOMA-B	125.2 (72.1–214.1)	76.3 (42.6–133.1)	152.0 (97.1–248.3)	<0.001
Cholesterol (mmol/L)	5.10 (4.38–5.80)	4.79 (4.01–5.44)	5.26 (4.66–5.98)	<0.001
LDL (mmol/L)	3.12 (2.49–3.76)	2.75 (2.16–3.39)	3.30 (2.70–3.93)	<0.001
HDL (mmol/L)	1.09 (0.91–1.35)	1.04 (0.85–1.27)	1.11 (0.93–1.37)	<0.001
Triglycerides (mmol/L)	1.59 (1.23–2.18)	1.73 (1.31–2.37)	1.55 (1.20–2.09)	<0.001
AST (μkat/L)	0.35 (0.28–0.48)	0.35 (0.28–0.50)	0.35 (0.30–0.48)	0.8
ALT (μkat/L)	0.45 (0.32–0.70)	0.43 (0.32–0.70)	0.45 (0.32–0.70)	0.8
γGT (μkat/L)	0.48 (0.32–0.75)	0.50 (0.33–0.87)	0.47 (0.30–0.72)	0.07
ALP (μkat/L)	1.64 (1.14–3.02)	1.25 (1.04–1.65)	2.27 (1.25–3.35)	<0.001
CRP (nmol/L)	5.71 (2.86–10.48)	5.71 (2.86–11.43)	5.71 (3.81–10.48)	0.5
Fibrinogen (μmol/L)	11.64 (10.17–13.38)	11.44 (9.91–13.17)	11.76 (10.29–13.52)	0.04

HbA1c levels in Ob-Eu refer to measurement in a subgroup of 290 patients. Data are expressed as medians (with interquartile range in parentheses). Comparison between populations was performed by Mann–Whitney test on log-transformed data and χ^2^ test

*BMI* body mass index; *REE* resting energy expenditure; *HOMA-IR* homeostatic model of insulin resistance; *HOMA-B* homeostatic model of β cell function; *LDL* low density lipoprotein; *HDL* high density lipoprotein; *AST* aspartate aminotransferase; *ALT* alanine aminotransferase; *γGT* gamma glutamiltransferase; *ALP* alkaline phosphatase; *CRP* C-reactive protein

**Fig. 1:**
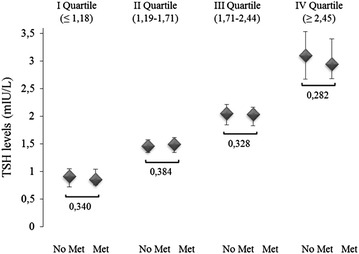
Comparison of TSH quartiles between obese euthyroid obese patients with diabetes and treated with metformin (Met) and those without diabetes and not treated with metformin (No Met). Medians (*diamonds*) and interquartile ranges (*lines*) are displayed

Analysis of thyroid function showed a mild increase in TSH levels in a subset of 30 patients (5.6 %). None of these harbored any increase in anti-thyroid antibody titer or abnormal free thyroid hormone levels. Table 1 illustrates similar TSH levels between metformin-treated diabetic patients and metformin-untreated subjects. Based on the TSH reducing effects previously found to be elicited by metformin, our attention was mainly focused on TSH values in metformin users and non-users, yet data dichotomization by median TSH levels o 1.72 mIU/L showed a comparable distribution of metformin users between top and bottom TSH bearers (24.3 and 23.2 %, respectively), with results being equivalent if TSH was stratified by quartiles (Fig. 1) or logistic regression analysis (Table 2). Oppositely, analysis of fT4 levels showed significantly higher fT4 levels in metformin-treated diabetic subjects compared to metformin-untreated ones. Interestingly, when a subset of 113 metformin-untreated obese patients with incident diabetes mellitus was considered separately, these patients exhibited lower TSH [1.50 (IQR 1.12–2.01) vs. 1.80 (IQR 1.24–2.51) mIU/L; p = 0.02] and greater abdominal obesity [waist, 132 (IQR 121–141) vs. 114.7 (IQR 100.4–132.5) cm; p < 0.001] than the normoglycaemic counterpart, devoid of differences in fT4 levels [11.1 (IQR 10.2–12.4) vs. 11.3 (IQR 10.2–12.6) pg/mL] or BMI values [44.5 (IQR 40.3–49.3) vs. 43.5 (IQR 39.5–48.2) kg/m^2^]. Gender stratification showed no differences in TSH and fT4 levels (data not shown).

**Table 2:** Logistic regression analysis on values of TSH and BMI dichotomized by median values (0 = bottom values; 1 = top values) in the population as a whole

Variables	TSH	BMI
Age	0.98 (0.96–0.99)	0.99 (0.98–1.01)
Female gender	1.08 (0.59–1.73)	*5.67 (3.61*–*8.90)*
TSH	–	1.05 (0.89–1.24)
BMI	0.97 (0.94–1.01)	–
REE	1.00 (0.99–1.00)	1.003 (1.002–1.003)
HOMA-IR	1.01 (0.99–1.02)	0.99 (0.98–1.01)
Use of metformin	1.09 (0.78–1.52)	1.29 (0.90–1.85)

Odds ratio and 95 % confidence intervals are displayed. *BMI* body mass index; *REE* resting energy expenditure; *HOMA-IR* homeostatic model of insulin resistance. Use of metformin was categorized as 0 = no use of metformin, 1 = use of metformin. Significant values are in italics

Bivariate regression analyses on the whole dataset depicted a significant negative correlation between TSH and fT4 levels (rho = −0.091, p = 0.005), with each being weakly correlated in opposite direction with BMI (TSH: rho = 0.078, p = 0.02; fT4: rho = −0.074, p = 0.02) and waist circumference (TSH: rho = 0.063, p = 0.06; fT4: rho = −0.066, p = 0.04). The relationship between TSH and fT4 persisted after controlling for age, BMI, gender, HOMA-IR and the use of metformin (r = 0.088, p = 0.008). No correlation was observed between TSH and age. In sub-group analysis, patients aged >70 year (103 cases, 10.8 %) showed comparable TSH levels with the remainders (1.68 mUI/L [IQR 1.25–2.46] vs 1.72 mUI/mL, [IQR 1.21–2.47]); the possibility that this represented a ‘survivor’ sample of patients was hinted by the finding of a slightly healthier metabolic profile when compared to the younger counterpart, i.e. lower levels of total and LDL cholesterol, triglycerides, and HbA1c (data not shown). When thyroid function was plotted against serum parameters of cardiovascular risk in the population as a whole, TSH was only associated with total cholesterol (rho = 0.080, p = 0.01). Of note, fT4 levels were inversely related to triglycerides (rho = −0.091, p = 0.005), insulin (rho = −0.079, p = 0.02) and HOMA-IR (rho = −0.083, p = 0.01), and directly to HDL-cholesterol (rho = 0.106, p = 0.001). In testing the relationship between thyroid function and cardio-metabolic variables after controlling for potential confounders (i.e. age, gender, BMI, incident diabetes mellitus, use of metformin, use of statins, smoking), only fT4 remained correlated with age, BMI and HDL-cholesterol, while the correlation with triglycerides approached statistical significance (Table 3). No relationship was seen between thyroid function and C-reactive protein, fibrinogen and liver function tests, but with alkaline phosphatase (TSH: rho = −0.098, p = 0.002; fT4: rho = 0.070, p = 0.03). Likewise, no association related thyroid function to REE and metabolic efficiency, as well as BIA-derived parameters of body composition (data not shown).

**Table 3:** Correlation analysis between thyroid function and phenotypic variables of interest in the obese group as a whole, after controlling for age, gender, BMI and use of metformin

Variables	TSH		fT4	
	r	p	r	p
Age	−0.05	*0.1*	−0.08	*0.01*
BMI	0.05	*0.09*	−0.09	*0.006*
Waist	0.04	*0.3*	0.02	*0.5*
Percent fat mass	0.09	*0.02*	0.07	*0.06*
Cholesterol	0.506	*0.09*	−0.02	*0.5*
HDL	0.03	*0.3*	0.13	*0.001*
LDL	0.04	*0.3*	−0.05	*0.1*
Triglycerides	0.05	*0.2*	−0.07	*0.06*
Insulin	0.01	*0.7*	−0.03	*0.3*
HOMA-IR	0.01	*0.8*	−0.04	*0.2*
HOMA-B	0.02	*0.5*	0.01	*0.7*
AST	0.03	*0.4*	−0.07	*0.06*
ALT	−0.003	*0.9*	−0.003	*0.9*
γGT	0.004	*0.2*	−0.03	*0.4*
Alkaline phosphatase	−0.13	*0.001*	0.09	*0.02*

Italic values indicate p-values

Underlined values indicate significance

Italic underlined values indicate statistically significant p-values

Age and BMI were omitted as covariates when measured as independent variables

In multivariate linear regression analysis, TSH was best predicted by fT4 (standardized β = −0.079, p = 0.015), total cholesterol (standardized β = 0.074, p = 0.02) and BMI (standardized β = 0.065, p = 0.04). Replacement of BMI with waist circumference did not reach statistical significance. When FT4 levels were tested as the dependent variable, they were predicted by TSH (standardized β = −0.84, p = 0.01), metformin-treated diabetes mellitus (standardized β = 0.087, p = 0.008), BMI (standardized β = −0.83, p = 0.012) and age (standardized β = −0.80, p = 0.015). When waist circumference replaced BMI, it also entered the regression equation (standardized β = −0.077, p = 0.021). HOMA-IR did not enter the regression equation both when TSH and fT4 levels were tested.

## Discussion

Obesity is conventionally regarded as the result of unbalanced calorie intake and impaired energy expenditure acting on a predisposed genetic setting. A common explanatory model encompasses the lipostatic regulation system, in which energy stores generate signals that are compared with targets encoded in the brain, and differences between these drive our food intake levels, activity patterns, and resting and active metabolisms [26]. Although obesity predisposes to hyperthyrotropinaemia and reductions in fT4 levels [1, 2], TSH levels can still provide a peripheral index of thyroid activity also in the obese state [27]. So far, analyses on the metabolic correlates of thyroid function in obesity have provided little evidence, or were conducted in small cohorts so as to draw definitive conclusions [1, 2]. In this regard, a potential confounder is constituted by the antidiabetic agent metformin, based on its ability to reduce TSH levels in subjects with hypothyroidism and in those with euthyroidism harboring normal-high TSH levels [11–15, 18–20]. Metformin acts as insulin-sensitizer in multiple ways, such as by decreasing hepatic glucose production through inhibition of the mitochondrial respiratory-chain complex 1, by activating the cellular metabolic sensor AMPK, by increasing glucagon-like peptide 1 (GLP-1) levels, as well as by inducing islet incretin receptor gene expression [28–31]. The exact mechanisms linking metformin effects to variations in pituitary TSH secretion are currently unknown, but possibly involve inhibition of hypothalamic AMPK, counteraction of central T3 effects, dopaminergic effects and/or inhibition of pituitary TSH secretion [11, 32, 33]. In the current study, first of all we documented comparable TSH levels between obese patients treated and not treated with metformin. This result was confirmed by multiple statistical approaches. Oppositely, a slight yet significant increase in fT4 levels was seen in metformin-treated subjects compared with their untreated counterpart. This latter finding contrasts with the observation that metformin leaves unaltered free thyroid hormone levels [34], and possibly reflects an effect played by obesity on thyroid hormone metabolism either mediated by type 2 deiodinase activity, TH binding to TBG, or thyroxine degradation. However, we do not exclude that such finding simply results from metabolic differences existing between subgroups, since metformin-treated subjects were slightly older and mildly more insulin resistant, showed a more central distribution of body fat and higher BMI compared to the metformin-untreated counterpart. Along this line of reasoning, the positive correlation between fat mass and fT4 found by others [35] supports the hypothesis that abdominal adiposity may increase free thyroid hormone. In addition, diabetes mellitus can increase per se fT3 inactivation to rT3 and decrease T4 conversion to T3 due to lower type 2 deiodinase activity [36], while metabolic syndrome can lead to higher fT4 levels in obese subjects [37]. Our obese patients with incident diabetes mellitus were found to harbor lower TSH compared with their metformin-untreated counterpart. In this subgroup, individual analysis found no potential case of subclinical dysfunction and no significant metabolic impact was documented in correlation analyses likely due to the sample dimension and the overall high rate of severe obesity in our study (nearly 80 %). Further investigations in larger datasets are warranted to verify the clinical significance of this observation.

Together, our results pinpoint potentially different metabolic roles for TSH and fT4 in the obese setting. In general, TSH was associated with increasing BMI and total but not HDL cholesterol levels. These associations disappeared after controlling for individuals’ confounders. Oppositely, fT4 levels showed an inverse relation with BMI, insulin, HOMA-IR, triglycerides, in conjunction with a direct association with HDL-cholesterol. fT4 associations with the metabolic phenotype remained significant after adjustment for potential confounders. This leads us to speculate that fT4 reflects better than TSH the cardiometabolic risk of obesity: according to this view, TSH levels mirror the obese phenotype, while fT4 levels act as a proxy of the metabolic status related to the obese phenotype. Previous studies in morbidly obese patients found an association of increasing TSH, and less significantly decreasing FT4, with insulin and insulin resistance [3, 38, 39], yet none was controlled for individual confounders. On the other hand, population studies related low fT4 more tightly to cardiometabolic indices (BMI, insulin resistance, lipid profile and intima media thickness) than high TSH levels [40–43]. In a Korean study on 6241 non diabetic euthyroid subjects, those with the lowest fT4 quartile had twice the risk for insulin resistance as compared to those in the highest quartile after adjustment for age, sex, metabolic, and life style factors [44]. Nevertheless, the NHANES 2007–2008 analysis on 3114 euthyroid healthy men and women found that only TSH levels, and to a lesser degree fT3, were correlated with BMI and waist circumference, while fT4 levels were not [8]. Likewise, the Asklepios Study on 2315 healthy euthyroid middle-aged men and women found that a higher fT3–fT4 ratio (index of conversion from fT4 to fT3) was predictive of an unfavorable metabolic profile [45]. Differences in prevalence and degree of obesity, fat partition, genetic background, and insulin sensitivity may contribute to explain the observed discrepancies.

In conclusion, fT4 levels appeared to be related to the overall metabolic phenotype of obese patients, while the metabolic impact of TSH was less prominent. Incident diabetes seems associated with a reduction in TSH results, yet further studies in larger samples of euthyroid obese patients are needed to detail the individual clustering of TSH and fT4 levels with metabolic features in this setting.

## Abbreviations

ALP: alkaline phosphatase
ALT: alanine aminotransferase
AST: aspartate aminotransferase
BIA: bio-impedance analysis
BMI: body mass index
CRP: ultrasensitive C-reactive protein
FPG: fasting plasma glucose
fT3: free triiodothyronine
fT4: free thyroxin
GGT: gamma-glutamyltranspeptidase
HbA1c: glycated hemoglobin
HDL: high-density lipoprotein
HOMA-B: homeostatic model of β cell function
HOMA-IR: homeostatic model of insulin resistance
IFCC: International Federation of Clinical Chemistry
IFG: impaired fasting plasma glucose
LADA: latent autoimmune diabetes of the adult
LDL: low-density lipoprotein
REE: resting energy expenditure
T2DM: type 2 diabetes mellitus
TSH: thyroid-stimulating hormone
